# Enhanced multi-omics analysis reveals a lncRNA signature with 12 RNA modifications to predict tumor heterogeneity and potential therapy in non-small cell lung cancer

**DOI:** 10.1007/s12672-025-03677-8

**Published:** 2025-10-14

**Authors:** Hua You, Mingyu Fan, Limei Yin, Feifei Na, Liting You

**Affiliations:** 1 Shanghai Jiao Tong University School of Clinical Medicine, Shanghai, 200001 China; 2 Lung Cancer Treatment Center, West China Hospital, Sichuan University, Chengdu, 610041 China; 3 Department of Health Management and Institute of Health Management, Sichuan Provincial People’s Hospital, University of Electronic Science and Technology of China, Chengdu, 610041 China; 4 Chinese Academy of Sciences Sichuan Translational Medicine Research Hospital, Chengdu, 610041 China; 5 Division of Thoracic Tumor Multimodality Treatment, Cancer Center, West China Hospital, Sichuan University, Chengdu, 610041 China; 6 Department of Laboratory Medicine, West China Hospital, Sichuan University, Chengdu, 610041 China

**Keywords:** RNA modification, Long non-coding RNA, Non-small cell lung cancer, Tumor heterogeneity, Potential therapy

## Abstract

This study delves into the landscape of RNA modification (RM)-related long non-coding RNAs (lncRNAs) within non-small cell lung cancer (NSCLC). We aim to uncover their significance in cancer biology and potential clinical implications. We utilized diverse datasets to identify 444 RM-related genes with 12 RMs. RM scores were computed, and associations with survival were analyzed. Weighted gene co-expression network analysis identified 730 RM-related lncRNAs. Univariate Cox regression identified 63 prognostically significant lncRNAs, leading to the classification of NSCLC samples into two clusters. Distinct differences in overall survival and disease-free interval were observed between the identified lncRNA clusters, showcasing their prognostic relevance. Molecular characterization uncovered mutation landscape variations, with cluster 2 displaying higher mutation rates in TP53 and TTN. Cluster-specific genomic alterations, immune cell infiltration, and immune checkpoint gene expression patterns were identified. Drug sensitivity analysis revealed distinct profiles, with cluster 1 showing potential resistance to a combined approach of certain chemotherapy and immunotherapy, while cluster 2 may be suitable for monotherapy with specific chemotherapeutic or targeted agents. In conclusion, this study stands as the first and most comprehensive exploration, elucidating the intricate connections between RM, lncRNAs, NSCLC, and tumor immunity. Its findings significantly enhance our comprehension of NSCLC heterogeneity, offering pivotal insights and paving the path toward personalized treatment strategies.

**Supplementary Information:**

The online version contains supplementary material available at 10.1007/s12672-025-03677-8.

## Introduction

Lung cancer is the leading cause of cancer death in individuals aged 50 and older [1]. Non-small cell lung cancer (NSCLC) is a prevalent and remarkably diverse subtype of lung cancer, accounting for more than 80% of all lung cancers [2]. Adding to the complexity, the responses to various therapies in different patients with NSCLC exhibit significant variability. NSCLC treatment research continued to focus on immunotherapy, targeted therapies, and personalized medicine to address the disparities and improve treatment outcomes for diverse populations [3]. Chemotherapy, while still important, is being improved with new formulations. Future directions include increased focus on multi-target therapies, as well as the integration of artificial intelligence to optimize treatment strategies [4]. Hence, the accurate identification of responders to chemotherapy, immunotherapy or targeted therapies holds the potential to streamline treatment, mitigate progression risks, and alleviate the overall disease burden.

The significant tumor heterogeneity and intricate interplay involving RNA modification (RM) have garnered increasing attention in cancer research due to their contribution to diverse responses [5, 6]. RM, which refers to the manipulation or alteration of RNA molecules involved in various cellular processes, plays pivotal roles in orchestrating diverse facets of RNA metabolism, contributing to the complexity of gene expression regulation [7, 8]. Specifically, among these RNA modifications, 5-methylcytosine (m5C) [9] involves the addition of a methyl group to the cytosine base, while 5-methyluridine (m5U) [6] entails the addition of a methyl group to the uridine base. In the case of 7-methylguanosine (m7G) [10], a methyl group is added to the guanosine base. Furthermore, alternative polyadenylation (APA) [11] induces changes in the 3' end polyadenylate chain length of RNA molecules, while tRNA modifications encompass various alterations at the uridine 34 position. N1-methyladenosine (m1A) [12] and N1-methylguanosine (m1G) [13] involve the addition of a methyl group at specific positions, whereas N6-methyladenosine (m6A) [14] occurs at the N6 position of the adenine base. Additionally, pseudouridine transforms uridine bases into pseudouridine, stabilizing RNA structures. RNA cap methylations pertain to methylation at the 5' end, and RNA editing involves the insertion, deletion, or substitution of bases in RNA molecules. Lastly, uridylation involves the addition of uridine, and 2'-O-methylation involves methylation at the 2'-hydroxyl group of RNA molecules. In the context of NSCLC, the role of RM holds vital significance [15]. Firstly, RNA molecules, particularly messenger RNA, assume a central role in the regulation of gene expression [16]. Modulating RNA can exert influence over the production of specific proteins associated with cancer cell growth, survival, and metastasis [17]. Moreover, RM plays a crucial role in the context of immunotherapy for NSCLC [18]. Ongoing studies explore the potential of RNA-based vaccines and immunomodulatory agents to stimulate the immune system, enhancing its ability to recognize and attack cancer cells more effectively. Furthermore, RM research significantly contributes to the identification of RNA-based biomarkers for NSCLC. Changes in the expression levels of specific RNAs can serve as diagnostic or prognostic indicators, facilitating the development of personalized treatment approaches [19, 20]. Thus, understanding how NSCLC cells develop resistance to existing treatments involves a comprehensive study of RM. This knowledge is crucial for designing strategies to overcome resistance and improve treatment outcomes, ultimately advancing precision medicine for NSCLC.

Long non-coding RNAs (lncRNAs), a class of RNA molecules longer than 200 nucleotides with limited protein-coding potential, have emerged as key players in cancer biology [21]. These molecules are involved in diverse cellular processes, and their dysregulation is closely associated with tumorigenesis and cancer progression [22]. Recent studies have implicated lncRNAs in mediating the effects of RMs on cancer development, making them potential therapeutic targets and prognostic markers [23, 24]. Notably, lncRNAs have been found to modulate immune responses, influence stromal components, and impact the overall tumor microenvironment. This multifaceted involvement of lncRNAs underscores their significance in shaping the NSCLC landscape. Therefore, delving into the depths of tumor heterogeneity and understanding the intricate crosstalk between RMs, lncRNAs, and NSCLC holds promise for unveiling novel therapeutic strategies and refining patient stratification [25].

In this study, we present the first and most comprehensive analysis of RM-related lncRNA subtypes in NSCLC based on 12 RMs, shedding light on their clinical relevance and potential impact on disease management. Our analysis begins by identifying RM-related genes and assessing their differential expression in NSCLC samples. Subsequently, weighted gene co-expression network analysis (WGCNA) is employed to identify lncRNA clusters associated with RM. We then investigate the clinical implications of these clusters, including their impact on patient survival, the tumor microenvironment, genomic alterations, and response to immunotherapy and chemotherapy. To date, our investigation stands as the most comprehensive analysis of RM-related lncRNA in NSCLC. Our primary objective is to offer a more profound comprehension of the molecular and genomic foundations underlying NSCLC, with the ultimate goal of identifying potential therapeutic targets for the development of personalized treatment strategies.

## Materials and methods

### Data acquisition

We obtained The Cancer Genome Atlas (TCGA) RNA-seq data, clinical phenotype and survival data from tumor samples via the University of California Santa Cruz (UCSC) Xena database (https://xenabrowser.net/hub/), and additional datasets from the Gene Expression Omnibus (GEO) repository (https://www.ncbi.nlm.nih.gov/geo/) using the GEOquery package. Probes were annotated using the AnnoProbe package. Gene expression data from the Kaplan‒Meier Plotter database (https://kmplot.com/analysis/) were utilized for gene signature validation. In our comprehensive investigation, we incorporated the 12 most prevalent RMs into our study, including m5C, m5U, m7G, APA, modifications of U34 on transfer RNA, m1A, m1G, m6A, pseudouridine, RNA cap methylations, RNA editing, uridylation, and 2'-O-methylation.

### Identification of RM-related genes and lncRNA

We performed differential expression analysis of RM-related genes between cancer and normal samples using the R package limma (v3.42.2) with a significance threshold of adj.P.Val < 0.05 and |log_2_FC|> 0.585. We computed sample-specific RM scores using the single-sample GSEA (ssGSEA) function from the GSVA package (v1.34.0), utilizing all RM-related genes. Subsequently, we categorized samples into high and low score groups based on the median score and plotted Kaplan–Meier survival curves for overall survival (OS), disease-free interval (DFI), disease-specific survival (DSS), and progression-free interval using the survival (v3.2–7) and survminer (v0.4.8) packages. Additionally, we utilized the ssGSEA function to calculate enrichment scores for 28 immune infiltrating cell types, followed by Spearman correlation analysis to assess the relationship between RM scores and immune cell enrichment scores. Furthermore, using the estimate package (v1.0.13), we computed Tumor Purity, Immune Score, Stromal Score, and ESTIMATE Score for cancer samples and conducted Spearman correlation analysis between RM scores and these scores. Information on immune checkpoint genes was sourced from PMID: 32814346 [26].

To identify RM-related lncRNAs, we ranked the lncRNA expression matrix for cancer samples based on mean expression values and selected the top 10,000 lncRNAs for constructing a network using the WGCNA package (v1.69) [27]. Modules were defined with a minimum module size of 50 genes and a mergeCutHeight of 0.1 [28, 29]. We selected modules exhibiting the highest correlation with RM scores, and lncRNAs within these modules were considered as RM-related lncRNAs for subsequent analysis.

### Unsupervised clustering

We conducted univariate Cox regression analysis on the identified key lncRNAs within the selected modules, incorporating OS data. LncRNAs with p < 0.05 were considered significantly associated with OS. Unsupervised clustering of cancer samples was performed based on the expression profiles of these prognostic lncRNAs using ConsensusClusterPlus package (v1.50.0). Subtype-specific enrichment scores for Hallmark gene sets were calculated using ssGSEA. We examined the variations in clinical features in clusters, including age, pathologic M stage, pathologic N stage, pathologic T stage, radiotherapy, gender, smoking history, tumor stage, and disease. Cox univariate regression analysis, which included other prognostic factors such as age, gender, and tumor stage, was performed to demonstrate the independent prognostic value of the risk score.

### Validation of RM-related signature

We conducted WGCNA analysis on the top 10,000 genes, including both protein-coding genes and lncRNAs, characterized by the highest mean expression values in GSE37745 [30] and GSE42127 [31]. Feature genes were identified by exploring modules with the most significant correlations with clusters in both datasets. These feature genes were subsequently utilized for cluster classification in GEO data, employing the CMScaller package (v0.99.1) [32]. To validate the prognostic significance of the identified clusters and reaffirm their stable prognostic value, we generated Kaplan–Meier survival curves.

### Molecular features

We employed the maftools package (v2.2.10) to visualize the mutation landscape of samples across clusters, utilizing TCGA-NSCLC mutation data. Significantly mutated genes were identified through Fisher’s test. Furthermore, we computed genomic alterations, encompassing fraction of genome alteration (FGA), fraction of genomic gained (FGG), and fraction of genome lost (FGL), utilizing TCGA-LUNG samples. Additionally, we calculated the number of focal loss, arm gain, focal gain, and arm loss, and used violin plots to visualize their distribution differences across clusters.

### Drug sensitivity

We investigated disparities in immune cell infiltration by leveraging sample-specific immune enrichment scores. We conducted statistical analyses to examine variations in the expression of immune checkpoint genes, antigen presentation capability, co-stimulatory, and co-inhibitory molecules. T-cell inflammatory signature (TIS) scores were calculated using 18 tumor inflammation signature genes obtained from PMID: 31684954 [33], and leukocyte fraction information was sourced from PMID: 29628290 [34]. Additionally, we evaluated and depicted HLA family gene expression differences in clusters. To predict the potential therapeutic response to immune checkpoint inhibitors (CTLA4 and PD1) for different clusters based on gene expression profiles, SubMap analysis was performed. Subtype-specific feature gene sets were utilized to compute enrichment scores within immunotherapy datasets, including GSE136961 [35]. Moreover, we predicted the response to 138 drugs (CGP2014) for each sample using the pRRophetic package (v0.5), resulting in the computation of half maximal inhibitory concentration (IC50) values.

### Statistical analysis

All analyses were conducted using R software (v4.3.1). To assess differences between two groups, Student’s t-test or the Wilcoxon test was employed, while the Kruskal–Wallis test was used to compare differences among multiple groups. A significance threshold of p < 0.05 was adopted to determine statistical significance.

## Results

### RM aberrations inducing tumor microenvironment heterogeneity in NSCLC

**Fig. 1:**
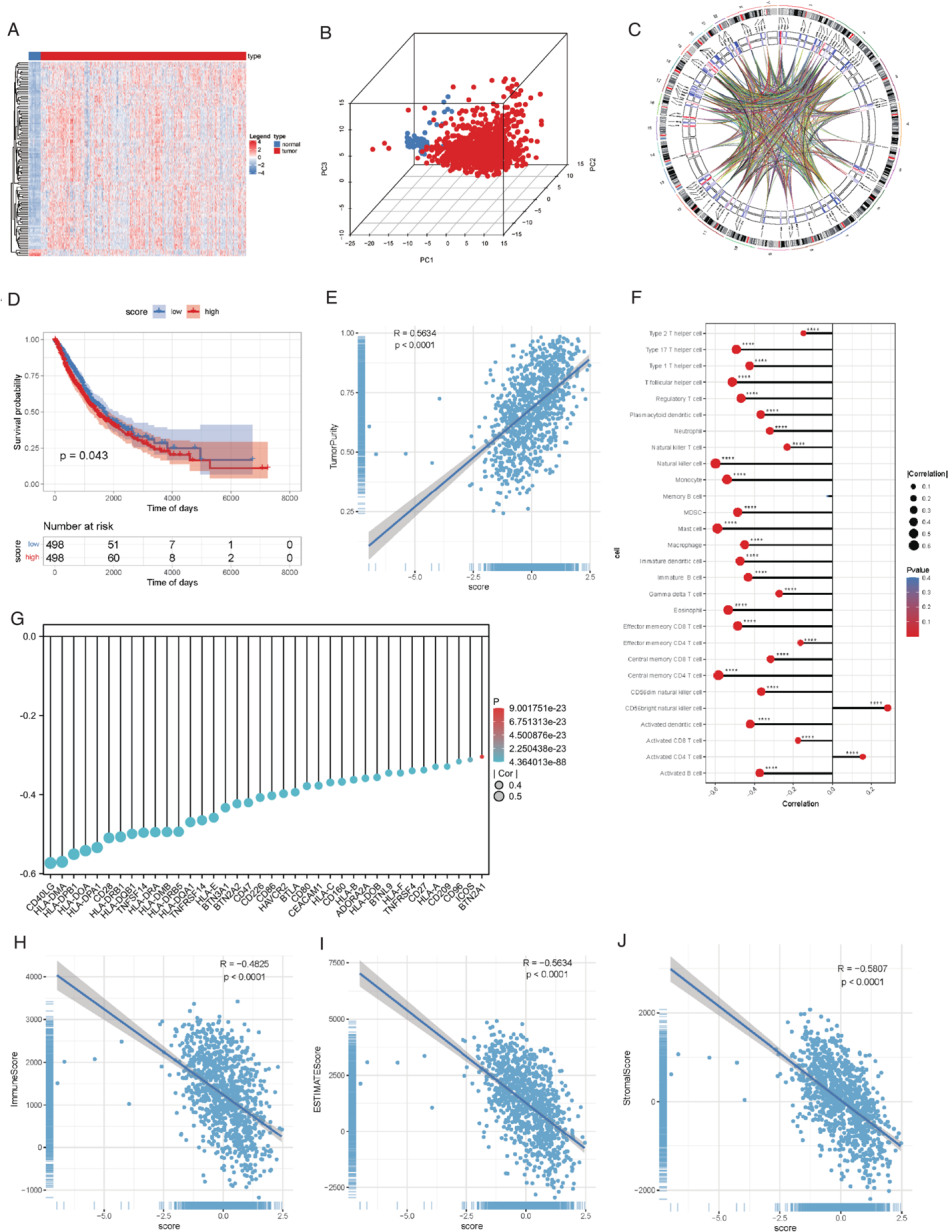
RM aberrations inducing tumor microenvironment heterogeneity in NSCLC. **A**–**C** Heatmap (**A**), PCA plot (**B**) and Circos plot (**C**) of the RM-related DEGs between NSCLC and normal lung tissues. **D** Kaplan–Meier analysis of NSCLC patients in high and low RM score groups based on 444 model genes. **E**–**J** Spearman correlation analysis between RM score with Tumor Purity (**E**), immune cell enrichment score (**F**), immune checkpoint gene expression (**G**), Immune Score (**H**), Stromal Score (**I**), and ESTIMATE Score (**J**). *RM* RNA modulation, *NSCLC* non-small cell lung cancer, *PCA* principal component analysis, *DEGs* differential expression genes. ****p < 0.0001

We retrieved RNA expression data and clinical information for 1,055 LUAD and LUSC samples from the UCSC Xena database (996 NSCLC samples and 59 adjacent normal samples), 196 NSCLC samples from GSE37745 [30], and 176 NSCLC samples from GSE42127 [31]. To focus our analysis on lncRNAs and protein-coding genes, we filtered the expression matrix to include only genes with a total expression sum greater than 1, resulting in 32,656 genes. Information on RM-related genes was gathered from various sources, including MODOMICS, MSigDB, RM2Target databases, and a relevant publication (PMID: 32300195 [5]), leading to a total of 444 RM-related genes for subsequent analysis (Table S1). Subsequently, we identified 115 differentially expressed RM-related genes based on predefined thresholds, comprising 111 upregulated and 4 downregulated genes (Table S2). These differentially expressed genes effectively discriminated between tumor and normal samples (Fig. 1A–C, Figure S1).

Using the set of 444 RM-related genes, we computed RM scores for cancer samples and subsequently classified patients into high and low-score groups based on the median score. Notably, these score-based groups demonstrated significant variations in OS and DSS, as depicted in Fig. 1D and Figure S2. At a microscopic level, we initially focused on tumor cells. We investigated the interplay between RM scores and Tumor Purity, revealing a substantial positive correlation (Fig. 1E). Moving beyond the tumor cells, our exploration extended to tumor immunity. We delved into the correlation between RM scores and immune cell enrichment scores. While memory B cells showed no notable correlation, other immune cell types exhibited significant associations with RM scores. Specifically, RM score demonstrated a negative correlation with the infiltration of various immune cell types, such as activated CD8^+^ T cells, effector memory CD8^+^ T cells, activated dendritic cells, and macrophages. In contrast, only activated CD4^+^ T cells and CD56 bright natural killer cells exhibited a positive correlation. This implies that a higher RM score is indicative of diminished immune infiltration (Fig. 1F, Table S3). Moreover, among the 67 immune checkpoint genes with available expression data [26], 38 exhibited noteworthy correlations with RM scores (p < 0.05, |cor|> 0.2). Notably, all genes demonstrated negative correlations. This includes the majority of activating checkpoint molecules such as CD80/86, CD28, CD40L, ICOS, etc. This may explain the poorer prognosis observed in patients with higher RM scores (Fig. 1G, Table S4). To gauge the overall impact, we further explored the relationship between RM scores and Stromal Score, Immune Score, and ESTIMATE Score, revealing significant negative correlations (Fig. 1H–J).

### Identification and clustering of prognostically significant RM-related lncRNA

**Fig. 2:**
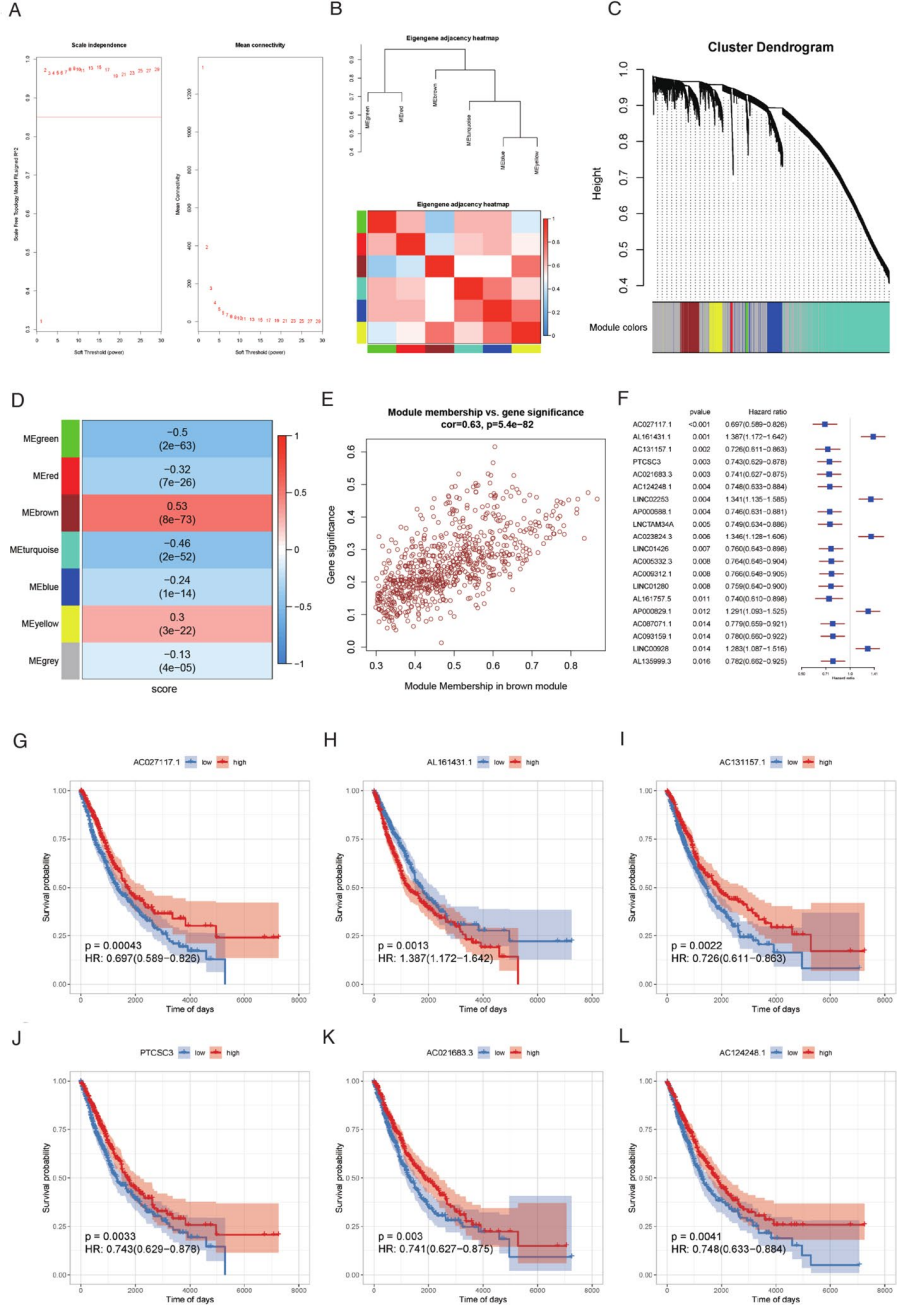
Identification of prognostically significant RM-related lncRNA. **A** Soft-thresholding results for module construction. **B** Correlation between modules. **C** Cluster dendrogram. **D** Correlation between modules and RM scores. **E** Relationship between module membership and gene significance in brown module. **F** Forest plot of the top 20 genes that independently impact OS by a univariate Cox regression. **G**–**L** Kaplan–Meier OS curves of the top 6 genes. *RM* RNA modulation, *lncRNA* long noncoding RNA, *OS* overall survival

LncRNAs play a crucial role in RM by actively participating in the intricate regulatory networks governing gene expression [36, 37]. To decipher the functional spectrum of lncRNAs, we employed WGCNA on the entire set of lncRNAs to pinpoint those associated with RM. This analysis revealed the identification of the brown module, demonstrating the strongest correlation with RM scores and encompassing 730 lncRNAs (Fig. 2A–E). Through univariate Cox regression analysis, we pinpointed 63 prognostically significant lncRNAs among the 730 module-related genes. Notably, AL161431.1 (HR = 1.387, 95%CI: 1.172–1.642), AC023824.3 (HR = 1.346, 95%CI: 1.128–1.606), and LINC02253 (HR = 1.341, 95%CI: 1.135–1.585), top3 high-risk lncRNAs, were correlated with poorer survival, while AC027117.1, AC131157.1, and AL161757.5 exhibited the opposite trend (Fig. 2F–L, Table S5). It has been reported that, with the aid of METTL3 (a key m6A writer), the lncRNA LINC02253 increases KRT18 mRNA stability and promotes cancer cells proliferation by activating the MAPK/ERK signaling pathway [38]. In our datasets, co-expression of LINC02253 and METTL3 was not been observed, while the positive correlations of LINC02253 with ADAD2, METTL1, METTL5, and METTL8 were significant (Figure S3, Table S6), suggesting that LINC02253 may stabilize METTL1 or other genes to modulate RM status and regulate NSCLC progression.

**Fig. 3:**
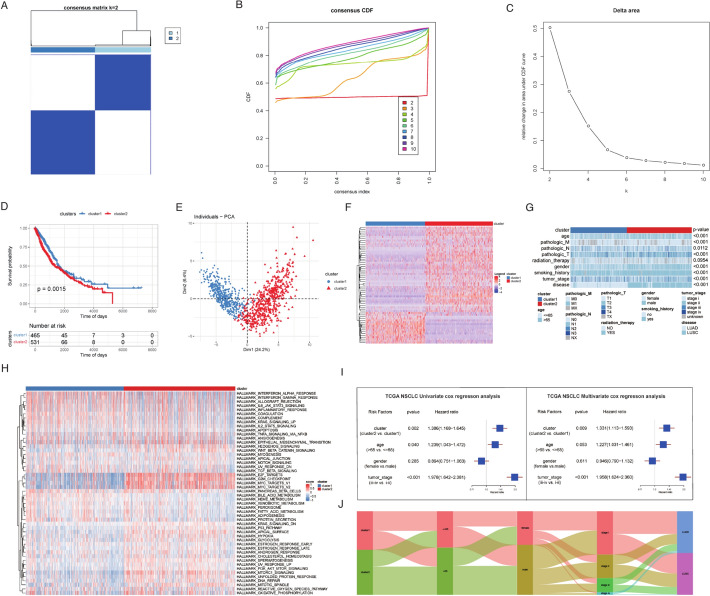
Unsupervised clustering of 63 RM‐related DEGs. **A** NSCLC patients in the TCGA cohort were grouped into two molecular clusters when k = 2 based on the 63 RM‐related DEGs. **B** The empirical cumulative distribution function plot of continuous k values (from 2 to 10). **C** Dendrogram of unsupervised consensus clustering. **D** Kaplan‒Meier analysis of OS among NSCLC patients in two different clusters in the TCGA cohort. **E** PCA plot of two clusters. **F** Heatmap of two clusters with expression profiles of prognostic lncRNAs. **G** Differences in clinical features between two clusters. **H** Heatmap of HALLMARK pathways between two clusters. **I** Univariate and multivariate Cox regression analysis for the clinical characteristics and lncRNA subtype in the TCGA NSCLC cohort. **J** Sankey diagram showed the relationship between two clusters and clinical features of NSCLC patients. *RM* RNA modulation, *DEGs* differential expression genes, *NSCLC* non-small cell lung cancer, *OS* overall survival, *PCA* principal component analysis, *lncRNA* long noncoding RNA

Unsupervised clustering using these 63 lncRNAs resulted in the classification of samples into two clusters (Fig. 3A–C). Both clusters exhibited significant differences in OS and DFI (Fig. 3D, Figure S4B–D). The lncRNAs displaying noteworthy expression differences between the identified clusters (Fig. 3E, F, Figure S4A). To further investigate the disparities between the two clusters, we explored both clinical and biological attributes. Initially, we examined the variations in HALLMARK pathways. The heatmap analysis revealed significant differences in the enrichment of various HALLMARK pathways (Fig. 3H, Figure S5A). Moreover, we assessed the distribution differences in clinical features, including age, pathologic M stage, pathologic N stage, pathologic T stage, radiation therapy, gender, smoking history, tumor stage, and disease, between the clusters. The results indicated significant differences in various clinical characteristics, with the exception of radiation therapy (Fig. 3G). Notably, the lncRNA clusters were found to be independent prognostic factors for NSCLC, demonstrating robust prognostic value along with the tumor stage (Fig. 3I, J).

### Construction and validation of a RM-related signature

**Fig. 4:**
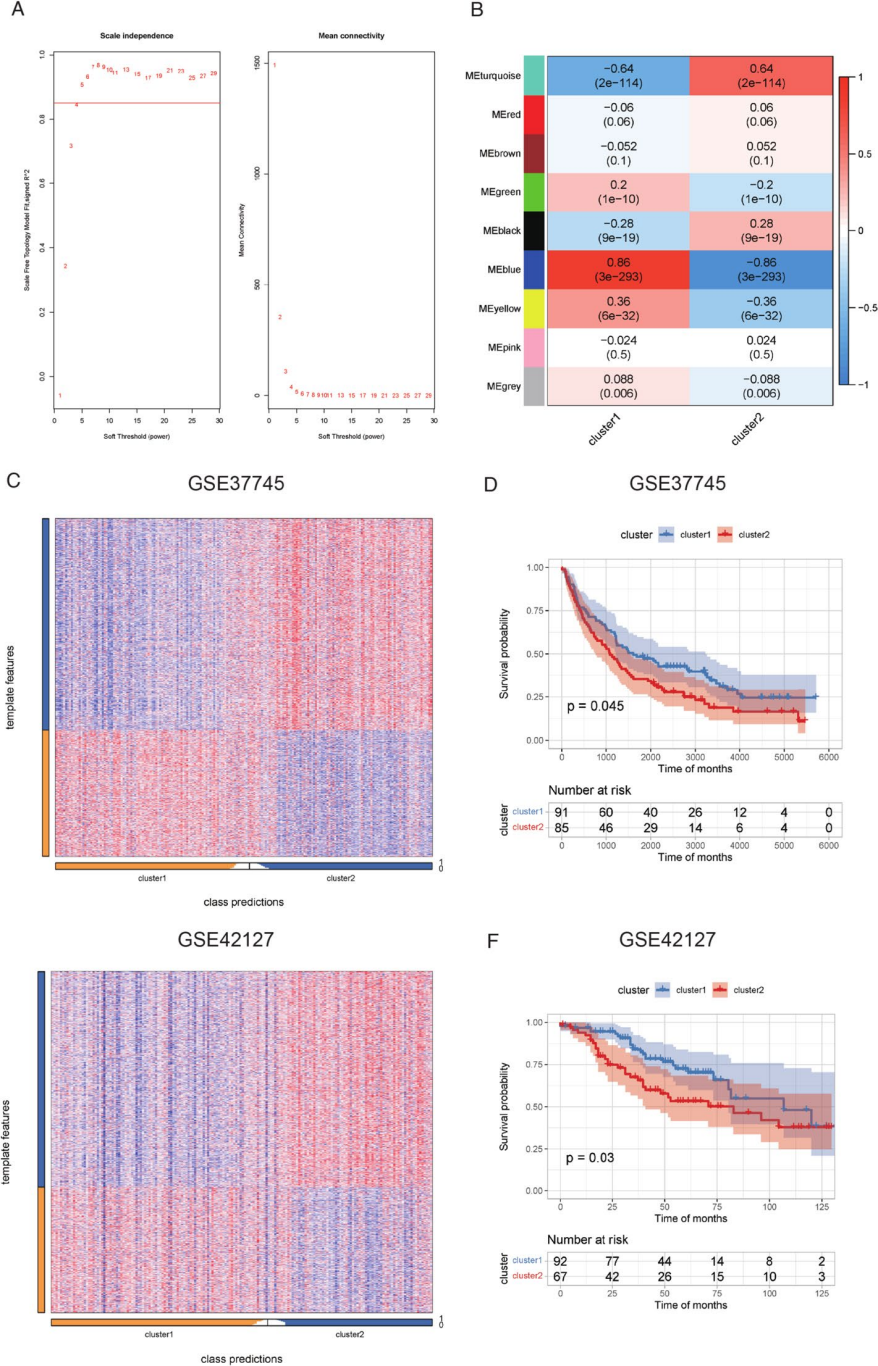
Identification and validation of RM-related gene signature in two clusters. **A** Soft-thresholding results for module construction. **B** Correlation between modules and two clusters. **C** Cluster identification in cohort GSE37745. **D** Kaplan‒Meier analysis of OS in two different clusters in cohort GSE37745. **E** Cluster identification in cohort GSE42127. **F** Kaplan‒Meier analysis of OS in two different clusters in cohort GSE42127. *RM* RNA modulation, *OS* overall survival

To identify feature genes, we employed the WGCNA approach. Using the expression matrix encompassing all genes, we applied a screening threshold of R^2^ > 0.85, which resulted in a power value of 5. Subsequently, we constructed a network using a one-step approach with a power value of 5, and modules were merged using a height threshold of < 0.1. This comprehensive process generated eight distinct modules. Among these, the blue module exhibited the most robust positive correlation with cluster 1 and included a total of 1638 genes. Conversely, the turquoise module displayed the strongest positive correlation with cluster 2 and encompassed 1976 genes. Further refinement involved filtering genes within these modules based on a gene significance value greater than 0, ultimately leading to the identification of 879 genes as cluster 1 feature genes and 1473 genes as cluster 2 feature genes (Fig. 4A, B, Table S7). To validate the lncRNA clusters identified by WGCNA, similar analyses were performed on external datasets (GSE37745 and GSE42127). Notably, cluster 1 and cluster 2 exhibited distinct separation (Fig. 4C, E), with cluster 1 displaying better survival than cluster 2, indicating statistically significant differences (GSE37745: p = 0.045, GSE42127: p = 0.03) (Fig. 4D, F). These results underscore the prognostic significance of the identified clusters, thereby affirming their stability.

### Heterogeneity of molecular characteristics and tumor immune microenvironment between RM clusters

**Fig. 5:**
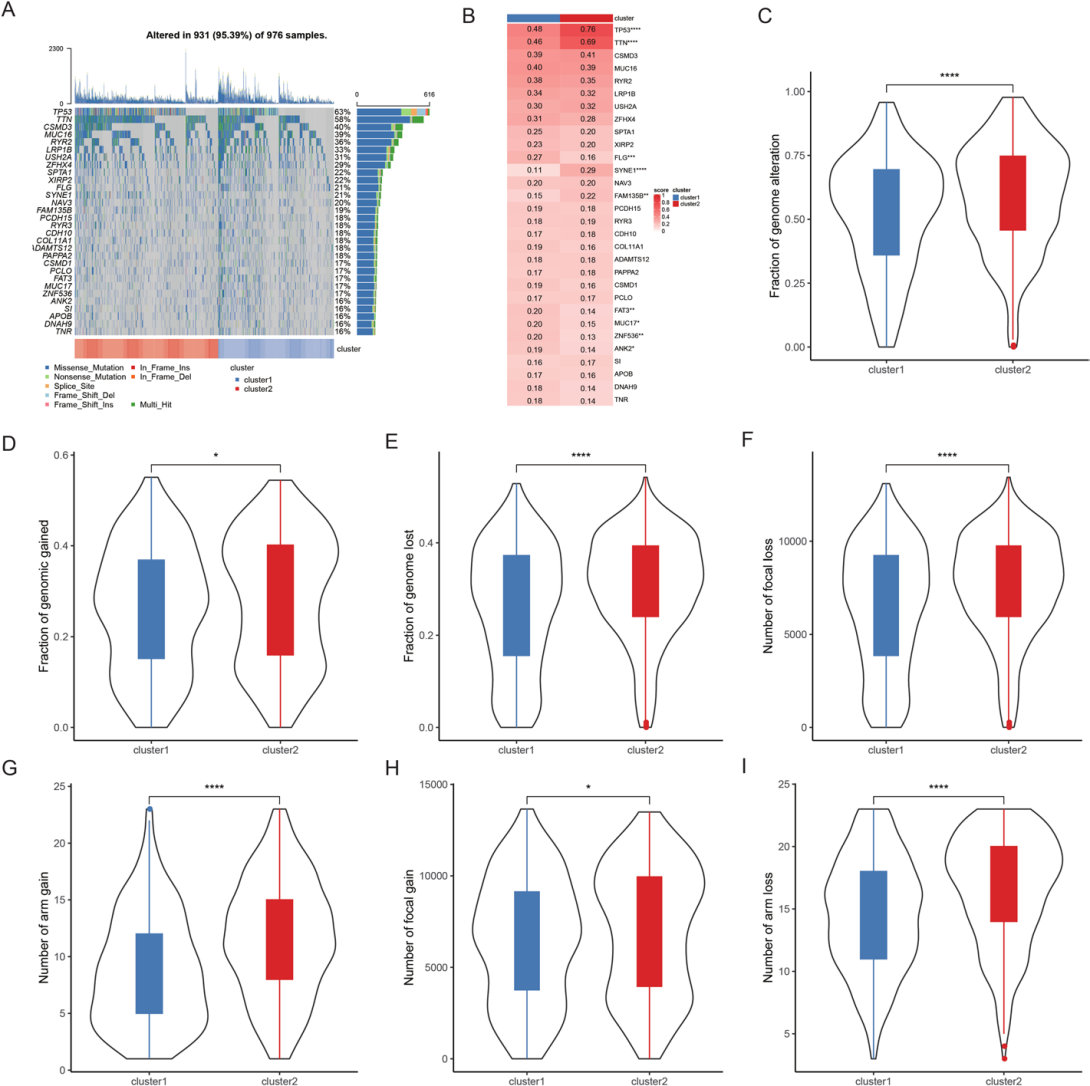
Molecular features between two clusters based on the RM-related signature. **A** Waterfall plot showing the top 30 mutated genes in two clusters. **B** Heatmap of gene mutation frequencies of top 30 mutated genes in two clusters. **C**–**E** Violin plot of fraction of genome alternation (**C**), genomic gained (**D**), and genome lost (**E**) in two clusters. **F**–**I** Violin plot of number of focal loss (**F**), arm gain (**G**), focal gain (**H**), and arm loss (**I**) in two clusters. RM, RNA modulation. ****p < 0.0001; ***p < 0.001; **p < 0.01; *p < 0.05

We visualized the mutation landscape and identified significantly mutated genes based on TCGA-NSCLC mutation data. It revealed that cluster 1 and cluster 2, characterized by heterogeneous RM profiling, exhibited significantly different molecular mutational characteristics. Notably, TP53 and TTN appeared as highly mutated genes in both clusters, but with significantly higher mutation rates in cluster 2 (Fig. 5A, B, Table S8), which was consistent with previous reports [39]. In addition, we scrutinized sample-level genomic alterations, including FGA, FGG, and FGL, finding that cluster 2 samples exhibited markedly higher scores for these alterations (Fig. 5C–E). Subsequently, we assessed patient chromosomal variations, encompassing arm gain/loss and focal gain/loss, revealing that cluster 2 patients displayed a higher level of chromosomal instability (Fig. 5F–I). These findings suggest that cluster 2 bears a higher mutational burden and increased invasiveness, thereby contributing to a poorer prognosis.

**Fig. 6:**
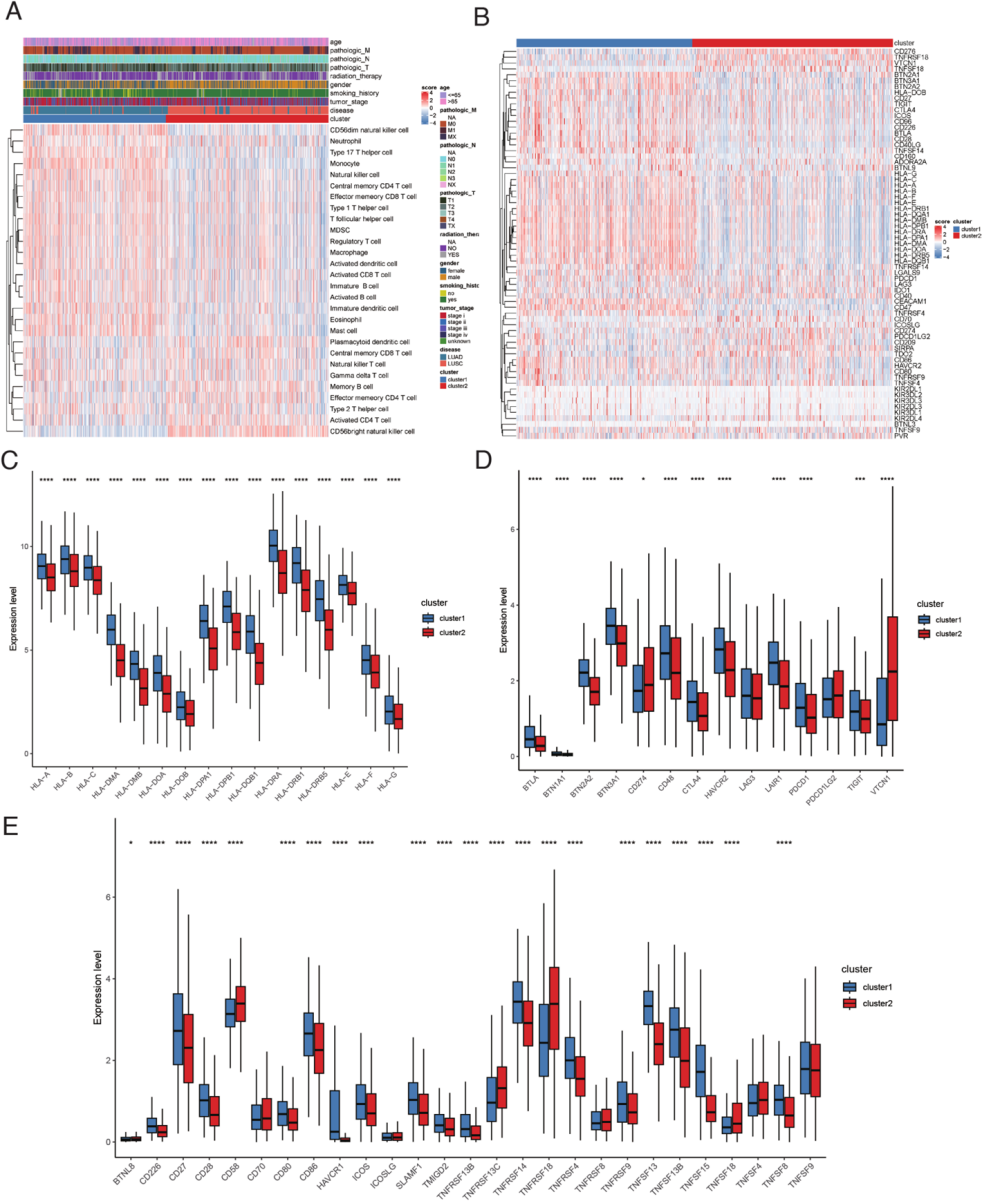
Deciphering the tumor immune microenvironment through an RM-related signature. **A** Heatmap of immune infiltration enrichment scores in two clusters. **B** Heatmap of immune checkpoint genes in two clusters. **C** Comparison of expression of HLA family genes in two clusters. **D** Comparison of co-stimulatory factors in two clusters. **E** Comparison of expression of co-inhibitory factors in two clusters. *RM* RNA modulation. ****p < 0.0001; ***p < 0.001; *p < 0.05

We assessed differences in immune cell infiltration between the clusters, revealing substantial variations. Cluster 1 samples exhibited higher enrichment scores for most immune cell types, such as activated CD8 + T cells, effector memory CD8 + T cells, and activated dendritic cells, while cluster 2 samples displayed higher scores for some certain immune cell subsets, such as central memory CD8 + T cells and plasmacytoid dendritic cells (Fig. 6A, Figure S5B). Analysis of immune checkpoint gene expression showed significant differences between the clusters, with most stimulated checkpoints exhibiting higher expression in cluster 1, such as CD28, ICOS and TNFSF14, implying potentially enhanced immunotherapy response (Fig. 6B). Furthermore, we observed elevated expression of HLA family genes in cluster 1, indicating an improved antigen presentation capability (Fig. 6C). Additionally, co-stimulatory and co-inhibitory genes, crucial for immunotherapy, displayed higher expression levels in cluster 1 (Fig. 6D, E). These findings indicate that cluster 2 may have a more immunologically “cold” tumor microenvironment, which could potentially contribute to the poorer prognosis observed in these patients.

### Prediction of chemotherapy and immunotherapy sensitivity via the RM-related signature

**Fig. 7:**
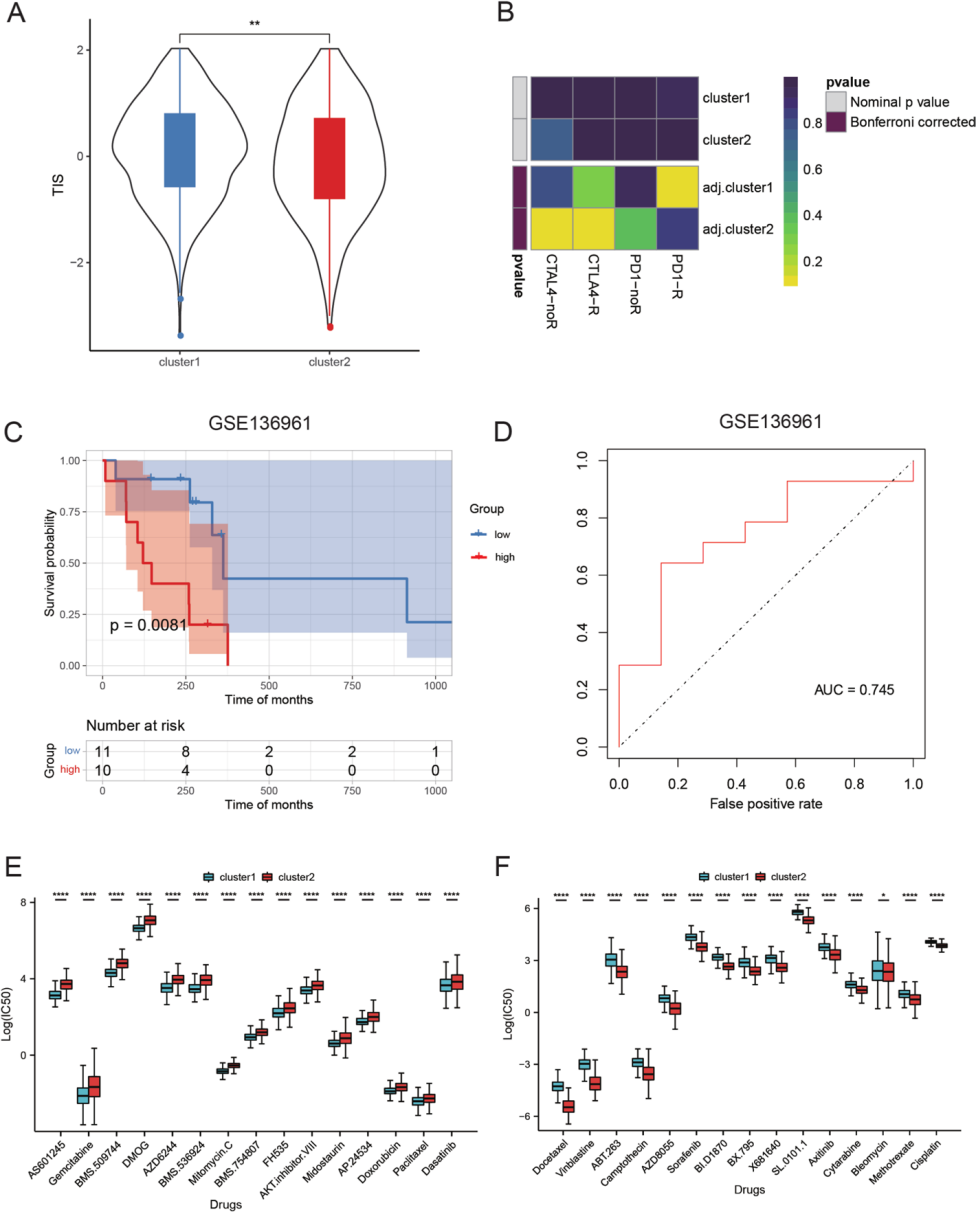
Prediction of chemotherapy and immunotherapy sensitivity via an RM-related signature. **A** Comparison of TIS in two clusters. **B** Submap analysis for prediction of immunotherapy outcomes in two clusters. **C** Kaplan‒Meier OS curves stratified by feature gene enrichment scores. **D** ROC curves based on gene enrichment scores. **E**, **F** Top 30 drugs with significantly higher IC50 in Cluster 1 (**E**) and Cluster 2 (**F**). *RM* RNA modulation, *TIS* T-cell inflammatory signature, *OS* overall survival, *ROC* receiver operating characteristic, *IC50* half maximal inhibitory concentration. ****p < 0.0001; **p < 0.01; *p < 0.05

TIS scores were higher in cluster 1, suggesting increased T-cell infiltration in the tumor microenvironment and a potentially better response to immunotherapy (Fig. 7A). SubMap analysis predicted a potentially better response to PD1 immune checkpoint inhibitors in cluster 1, although statistical significance was not achieved (p = 0.119) (Fig. 7B). Building upon the aforementioned results suggesting that cluster 1 samples may exhibit a better response to immunotherapy, we proceeded to analyze the feature genes from cluster 1. We obtained the GSE136961 dataset, which includes 21 samples subjected to anti-PD-1 immunotherapy. Among these samples, 9 demonstrated a durable clinical benefit (DCB), while 12 exhibited a non-durable benefit (NDB). We calculated the feature gene enrichment scores for each sample and subsequently divided them into groups based on the median enrichment score. Kaplan–Meier survival analysis was conducted, revealing significant differences in survival curves between the groups (Fig. 7C). The AUC value for predicting the immunotherapy response outcome using feature gene scoring reached 0.745 (Fig. 7D). Moreover, we predicted the response to 138 drugs and identified the top 30 drugs with significantly increased IC50 values in each cluster. Cluster 1 exhibited higher IC50 values for drugs like Bosutinib, Cisplatin, and Sorafenib, indicating potential resistance. In contrast, cluster 2 showed higher IC50 values for drugs including Bicalutamide, Camptothecin, and Vinblastine, suggesting resistance to these agents (Fig. 7E, F, Table S9). These findings imply that RM features hold the potential to guide clinical therapy decisions.

## Discussion

To our knowledge, our research stands out as the first to integrate a thorough examination of the 12 most prevalent RMs within the context of NSCLC and marks a pioneering and comprehensive exploration of the entire landscape of RM-related lncRNAs in NSCLC. This innovative approach, coupled with an inclusive consideration of immune-related factors, distinguishes our study as a trailblazer in the field. Our findings carry significant implications for unraveling the role of RM in NSCLC, providing valuable insights that pave the way for novel avenues in clinical intervention.

We observed significant dysregulation of RM-related genes in NSCLC samples compared to normal samples. This finding aligns with previous reports highlighting the involvement of RMs in cancer pathogenesis [40]. The identified differentially expressed RM-related genes effectively discriminated between cancer and normal samples, underscoring their potential diagnostic utility. The observed association between high RM scores and poorer OS and DSS in patients with NSCLC unveils a critical aspect of tumor biology. It implies that RM profiles can serve as independent prognostic indicators, holding potential for precise patient stratification and personalized treatment strategies.

The correlation between RM scores and the tumor microenvironment is a significant discovery [41]. The distinct immune phenotypes observed between clusters may arise through RM-lncRNA-mediated regulation of key immunological processes. Drivers of the "hot" microenvironment in cluster 1 consist of enhanced antigen presentation, T-cell activation, and immune cell recruitment. We observed elevated HLA genes (HLA-A/B/C/DMA/DMB/DOA/DOB; Fig. 6C) in cluster 1 may result from RM-lncRNA stabilization of CIITA (master HLA regulator) mRNA via m6A modifications, amplifying dendritic cell (DCs) maturation. Enriched DCs may induce activation of CD8 + T cells and other immune cell recruitment. In contrast, the mechanisms underlying the "cold" phenotype in cluster 2 may involve suppressed antigen presentation (downregulated HLA genes) and exhausted T cells (low checkpoint/co-stimulatory gene expression). However, various components of the TME, such as immune cells, cytokines, and tumor-associated factors, influence the efficacy of anti-tumor effects and immunotherapy [42–44]. Our findings still fail to elaborate the intricate crosstalk between RMs and immune cells in the tumor microenvironment highlights the multifaceted nature of cancer biology.

The identification of two distinct molecular clusters based on RM-related lncRNA expression opens up new avenues for personalized medicine. These clusters exhibited significant differences in OS and DFI. The observed differences in HALLMARK pathway enrichment and clinical features between clusters further emphasize the need for cluster-specific treatment strategies. These clusters not only differ in terms of clinical outcomes but also exhibit unique patterns of genomic alterations. This suggests that tailored treatment strategies based on these clusters may lead to more effective therapeutic interventions. Importantly, the identified lncRNA clusters were found to be independent prognostic factors, highlighting their potential clinical relevance. LncRNAs play a significant role in regulating RM-related gene expression through various mechanisms. They can modulate mRNA stability, regulate miRNA activity, affect splicing, modify chromatin structure, and even influence transcription and translation by interacting with DNA, RNA, and proteins [45, 46]. Among the identified lncRNAs through our multi-omics analysis, lncRNA PTCSC3 as a key regulator linking RNA modifications to NSCLC progression [47]. Mechanistically, PTCSC3 may stabilizes m6A writers via ceRNA networks. For example, PTCSC3 could capture miR-186-5p to increase METTL3 expression, enhancing m6A deposition [48]. However, the mechanisms by which the identified lncRNAs interact with RNA modifications to regulate NSCLC progression remain unclear, which is also the focus of our future research.

Our study highlighted differences in immunotherapy and chemotherapy response patterns between clusters. Understanding these resistance patterns may guide the selection of more effective therapy regimens tailored to individual patients. The observed differences in immune cell infiltration, immune checkpoint gene expression, and antigen presentation capabilities between clusters have significant implications for therapy response. Therefore, it can be presumed that cluster 1 is more sensitive to a combined approach of certain chemotherapy and immunotherapy, while cluster 2 may be suitable for monotherapy with specific chemotherapeutic or targeted agents. While our RM signature demonstrates robust prognostic and predictive value, its clinical implementation requires careful consideration of practical constraints. Translating these findings faces key limitations: clinical adoption of RM-related lncRNA signature as a diagnostic tool necessitates development of cost-effective detective assays (e.g., RT-qPCR panels, customized NanoString multiplex assay, or targeted RNA-seq for core lncRNAs), as well as prospective validation in clinical trials with treatment-response annotations.

It is important to acknowledge certain limitations in our study. Firstly, the retrospective nature of our analysis may introduce inherent biases. Secondly, abundant stromal components within the tumor microenvironment may introduce confounding biases in computational immune infiltration analyses and tumor purity estimates, potentially impacting the interpretation of our immune-related findings. Besides, the functional roles and molecular mechanisms of the identified RM-related lncRNAs were not experimentally validated, warranting further investigation. Additionally, external validation in larger and more diverse patient cohorts is needed, or advanced bioinformatics and artificial intelligence methods (such as generative adversarial networks [49]) should be used to increase our sample size, to confirm the clinical relevance of our findings.

## Conclusions

In conclusion, our study provides a comprehensive analysis of RM-related lncRNAs in NSCLC, shedding light on their roles in tumor heterogeneity, clinical outcomes, and potential therapeutic strategies. The identification of molecular clusters and their associations with clinical outcomes and therapeutic responses represents a step toward personalized medicine in NSCLC. Further validation and functional studies are warranted to fully exploit the clinical potential of RM-related lncRNAs in NSCLC management.

## Supplementary Information

Supplementary Material 1.

Supplementary Material 2.

## Data Availability

The datasets analyzed during the current study are available in The Cancer Genome Atlas (TCGA), the UCSC Xena database (https://xenabrowser.net/hub/), and the Gene Expression Omnibus (GEO) repository (https://www.ncbi.nlm.nih.gov/geo/).
