# Supplementary material for: Enhanced multi-omics analysis reveals a lncRNA signature with 12 RNA modifications to predict tumor heterogeneity and potential therapy in non-small cell lung cancer
Source: Discov Oncol. 2025 Oct 14;16:1873. doi: 10.1007/s12672-025-03677-8 (PMC12521086; doi:10.1007/s12672-025-03677-8)

Figure S1 Heatmap (A) and PCA plot (B) of the RM-related DEGs between NSCLC and normal lung tissues.

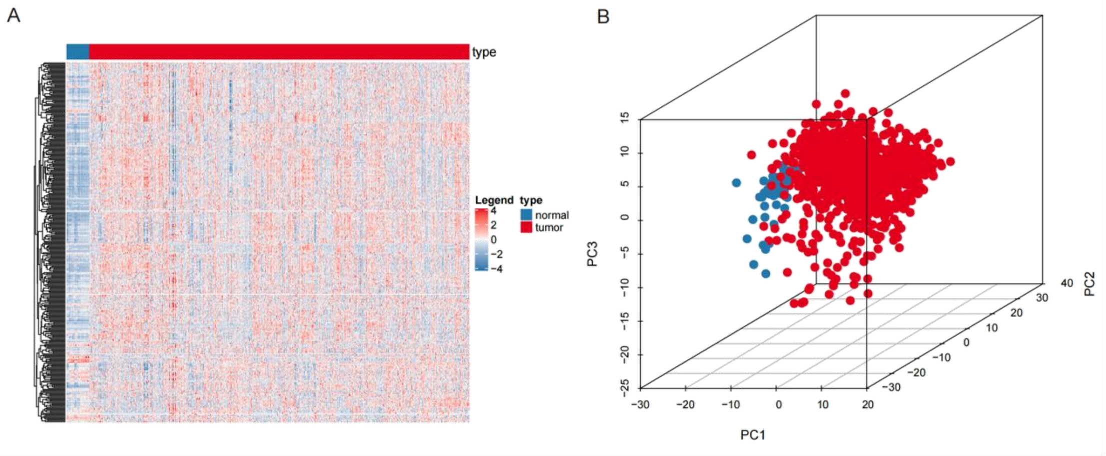

PCA, principal component analysis; RM, RNA modulation; DEGs, differential expression genes; NSCLC, non-small cell lung cancer.

Figure S2 Kaplan-Meier analysis of NSCLC patients in high and low RM score groups based on 444 model genes.

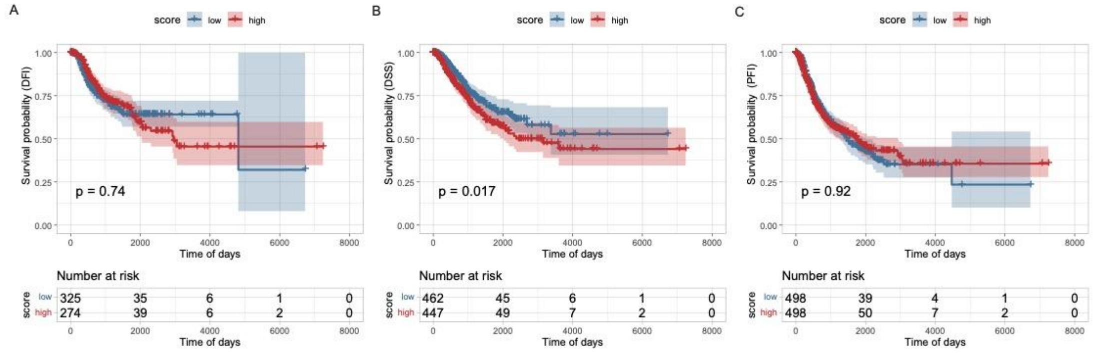

NSCLC, non-small cell lung cancer; RM, RNA modulation.

Figure S3. Spearman correlation analysis of LINC02253 and RM genes.

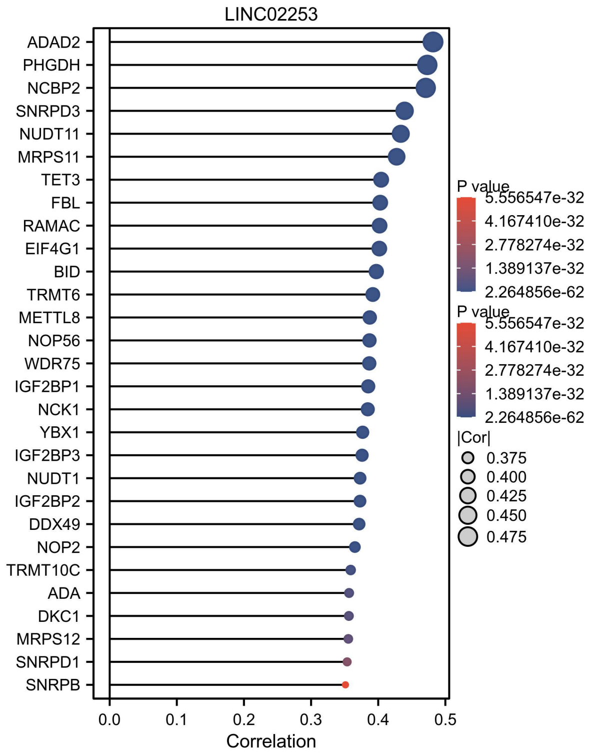

Figure S4. Expression level (A) and Kaplan–Meier analysis among NSCLC patients in two different clusters in the TCGA cohort (B-D).

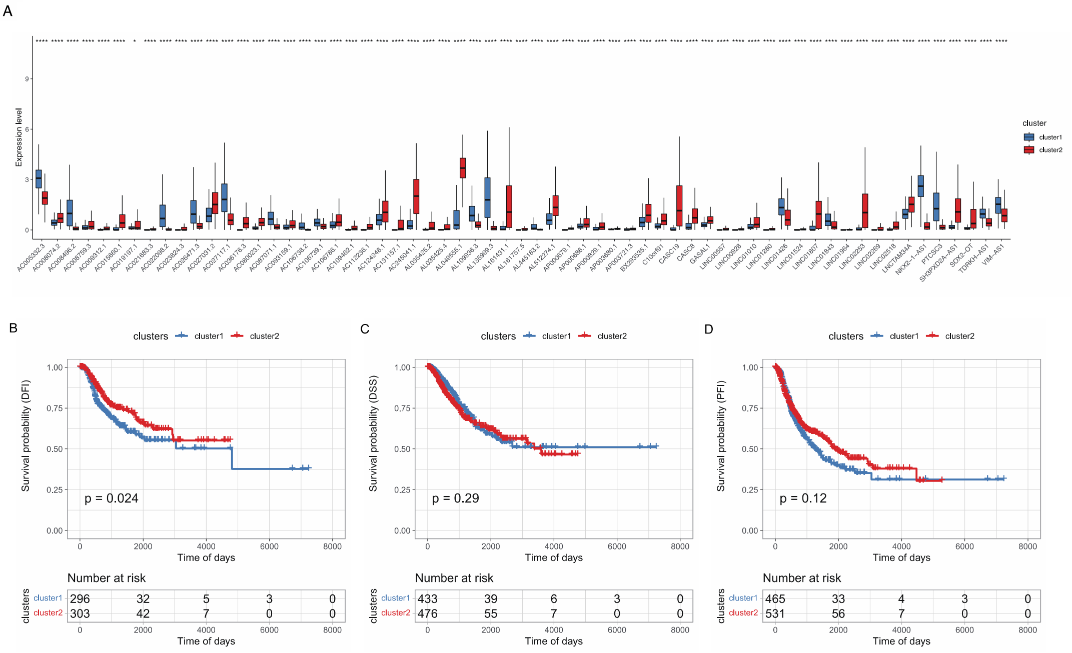

NSCLC, non-small cell lung cancer.

Figure S5. HALLMARK pathways (A) and immune infiltration GSVA scores (B) between two clusters.

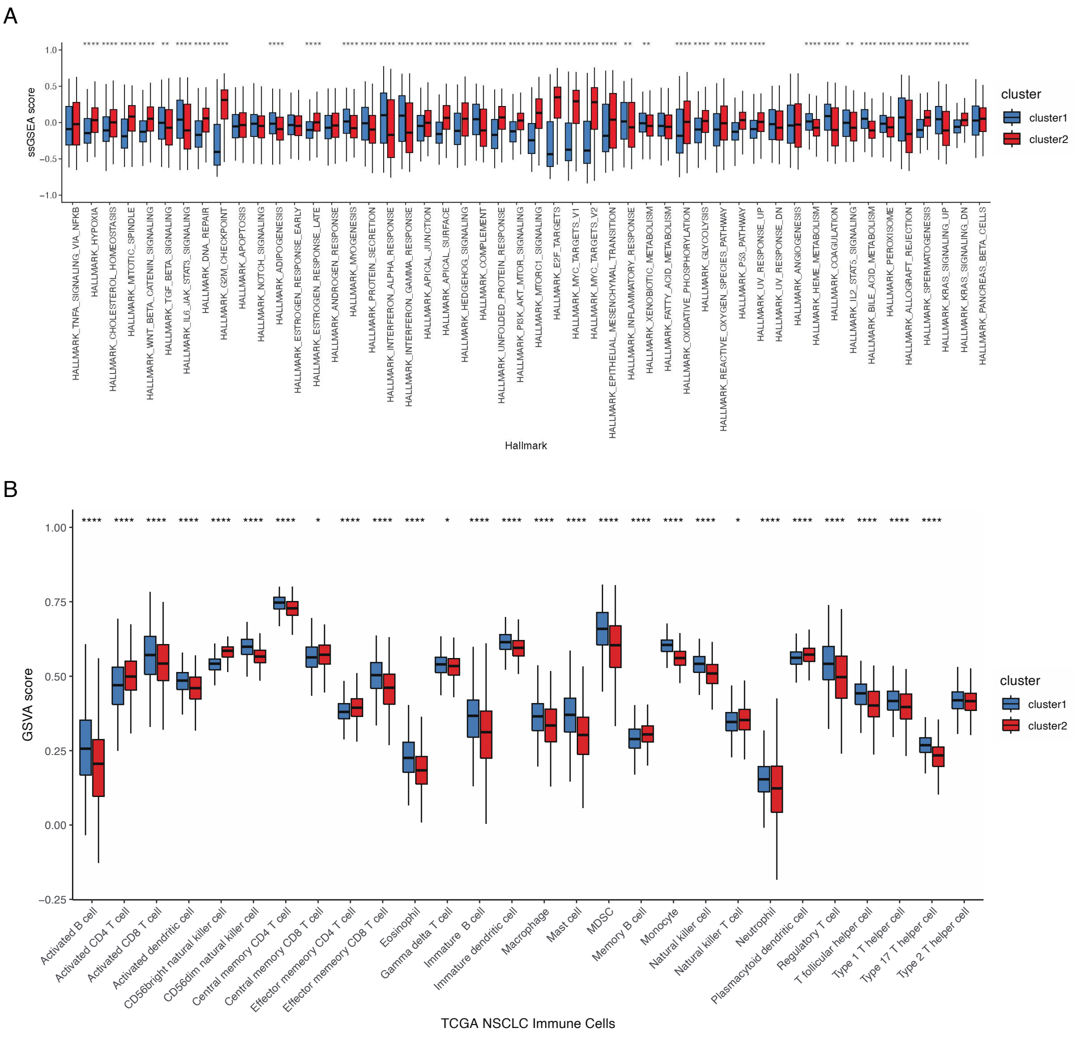

Supplement: Supplementary file 1 — Supplementary Material 1. [file 12672_2025_3677_MOESM1_ESM.pdf]
